# Recruitment of a Diverse Research Cohort in a Large Metropolitan Area for Dementia Intervention Studies

**DOI:** 10.1093/geroni/igaa057.847

**Published:** 2020-12-16

**Authors:** Melissa Reuland, Deirdre Johnston, Inga Antonsdottir, Morgan Bunting, Quincy Samus

**Affiliations:** 1 Johns Hopkins University School of Medicine, Baltimore, Maryland, United States; 2 Johns Hopkins Hospital, Baltimore, Maryland, United States; 3 Johns Hopkins School of Nursing, Baltimore, Maryland, United States; 4 Johns Hopkins University, Halethorpe, Maryland, United States; 5 Johns Hopkins University, Baltimore, Maryland, United States

## Abstract

In the near future, the costs, both human and financial, of dementia care will grow exponentially. Over five and a half million older Americans are estimated to be living with Alzheimer’s disease and related dementia (ADRD). By 2050, this is expected to increase to over 13 million, with persons of color being at the highest risk for developing dementia. Considerable federal, state and private funds have been committed to research to prevent, treat, and care for persons at risk for ADRD. However, enrollment of research participants, particularly those coming from diverse backgrounds into studies, is a perennial challenge and has serious implications. Between 2014 and 2019, a Johns Hopkins study team implemented a wide ranging research recruitment effort in the Baltimore-Washington DC area to enroll participants into two large federally funded dementia care coordination trials. A total of 2,063 study participants were self- or caregiver referred to these projects via referrals from organizations (e.g. religious, health, social service, aging, Medicaid clams) and targeted community outreach (e.g. events, media). Ultimately, 647 ADRD/study partner dyads were enrolled (31%). Outreach and recruitment challenges included stigma, lack of confirmed diagnosis, mistrust of research, and situational crises. The study team adapted enrollment criteria as challenges emerged, and ultimately spent $101,058 on outreach and recruitment to enroll 647 participant dyads. This represents a cost of $156.19 per dyad. This poster will provide background on the research program, detail the comprehensive outreach and recruitment strategies employed and their costs, and discuss best practices for recruiting this population.

